# Predictive Value of Diaphragm and Lung Ultrasonography for Weaning Failure in Critically Ill Patients with Acute Respiratory Failure Due to COVID-19 Pneumonia

**DOI:** 10.3390/diagnostics14202263

**Published:** 2024-10-11

**Authors:** Camila Fonseca, Claudio Novoa, Matias Aguayo, Ricardo Arriagada, Cristóbal Alvarado, César Pedreros, David Kraunik, Camila M. Martins, Patricia R. M. Rocco, Denise Battaglini

**Affiliations:** 1 Unidad de Paciente Crítico Adulto, Hospital Las Higueras, Talcahuano 4270918, Chile; camila.fonseca.p@gmail.com (C.F.); claudio.novoa09@gmail.com (C.N.); matias.aguayo.n@gmail.com (M.A.); rarrigarri@gmail.com (R.A.); cristobal.alvaradol@redsalud.gob.cl (C.A.); cesar.pedreros.saez@gmail.com (C.P.);; 2 Escuela de Kinesiología, Universidad Nacional Andrés Bello, Sede Concepción, Concepción 8370146, Chile; 3 Escuela de Kinesiología, Universidad San Sebastián, Sede Tres Pascualas, Concepción 7510602, Chile; 4 Facultad de Medicina, Universidad Católica de la Santísima Concepción, Concepción 4030000, Chile; 5 Unidad de Investigación, Hospital Las Higueras, Talcahuano 4270918, Chile; 6 Facultad de Medicina, Universidad de Concepción, Concepción 4070386, Chile; 7 AAC&T Research Consulting LTDA, Curitiba 82620-045, Brazil; 8 Laboratory of Pulmonary Investigation, Carlos Chagas Filho Institute of Biophysics, Federal University of Rio de Janeiro, Centro de Ciências da Saúde, Avenida Carlos Chagas Filho, 373, Bloco G-014, Ilha Do Fundão, Rio de Janeiro 21941-598, Brazil; prmrocco@gmail.com; 9 Department of Surgical Sciences and Integrated Diagnostics (DISC), University of Genoa, 16132 Genoa, Italy; 10 Anesthesia and Intensive Care, IRCCS Ospedale Policlinico San Martino, 16132 Genoa, Italy

**Keywords:** COVID-19, lung ultrasound, mechanical ventilation, weaning, diaphragm

## Abstract

Background: This study analyzed weaning characteristics and assessed the association of clinical and ultrasonographic indices—maximum inspiratory pressure (MIP), rapid shallow breathing index (RSBI), peak flow expiratory (PFE), diaphragm-thickening fraction (DTF), diaphragm thickness (DT), diaphragm excursion (DE), diaphragm-RSBI (D-RSBI), and lung ultrasound (LUS) patterns—with weaning failure. Methods: This retrospective cohort study included critically ill COVID-19 patients aged 18 and older who had been on invasive mechanical ventilation for at least 48 h and undergoing weaning. Exclusion criteria included absence of ultrasound assessments, neuromuscular diseases, and chronic cardio-respiratory dysfunction. Results: Among 61 patients, 44.3% experienced weaning failure, 27.9% failed the spontaneous breathing trial (SBT), 16.4% were re-intubated within 48 h, and 28% required tracheostomy. Weaning failure was associated with prolonged ventilation (29 vs. 7 days, *p* < 0.001), extended oxygen therapy, longer ICU stays, and higher ICU mortality. These patients had higher pressure support, lower oxygenation levels, a higher RSBI, and a lower MIP. While PEF, DTF, DE, and D-RSBI showed no significant differences, both right and left diaphragm thicknesses and the inspiratory thickness of the left diaphragm were reduced in failure cases. LUS scores were significantly higher before and after SBT in the failure group. Bivariate analysis identified RSBI [OR = 1.04 (95% CI = 1.01–1.07), *p* = 0.010], MIP [OR = 0.92 (95% CI = 0.86–0.99), *p* = 0.018], and LUS [OR = 1.15 (95% CI = 0.98–1.35), *p* = 0.025] as predictors of weaning failure; however, these associations were not confirmed in multivariate analysis. Conclusions: Ultrasound provides supplementary information during weaning, but no definitive association between ultrasound indices and weaning failure was confirmed in this study.

## 1. Introduction

Mechanical ventilation (MV) is a life-saving treatment for patients with acute respiratory failure (ARF). During the coronavirus disease 2019 (COVID-19) pandemic, healthcare systems experienced an unprecedented demand for critical care beds and MV [1]. Consequently, optimizing weaning strategies has become essential [2]. Weaning from MV, which accounts for approximately 40–50% of the total duration of MV, should be initiated as early as possible, because prolonged MV is associated with increased complications and mortality [3,4]. The weaning process is complex and typically takes several weeks [3,5], with failure rates varying from 26% to 42%, depending on the population and criteria used [6,7]. Currently, several parameters are used to predict the success of weaning from MV, although their predictive accuracy is often limited and can be accompanied by significantly variability. Commonly used clinical tests include maximum inspiratory pressure (MIP) [8] and the rapid shallow breathing index (RSBI), which are favored for their practical application in daily clinical practice [9].

In pursuit of more objective and bedside techniques to predict weaning success, there is growing interest in using ultrasound imaging of the diaphragm and lungs. Ultrasonography is a valuable tool during the weaning process because it provides real-time, non-invasive imaging of anatomical structures, allowing for immediate assessment of heart, lung, and muscle function [2]. Recent advances in diaphragmatic ultrasonography have introduced measures such as end-expiratory diaphragmatic thickness (DT), which may indicate diaphragm atrophy and ventilator-induced diaphragm dysfunction (VIDD). Additionally, the diaphragmatic thickening fraction (DTF) reflects inspiratory effort, as measured by transdiaphragmatic or esophageal pressure swing [10]. Despite its potential, there is conflicting evidence regarding the effectiveness of DTF, diaphragmatic excursion (DE), and DT in predicting weaning success. Newer indices, such as the diaphragmatic rapid shallow breathing index (D-RSBI), have shown promise in improving prediction accuracy compared to traditional RSBI [11].

This study aims to describe the characteristics and outcomes of weaning from MV (success/failure) in patients with ARF due to COVID-19. Additionally, it seeks to assess the relationship between conventional clinical weaning indices (RSBI, MIP, peak expiratory flow) and ultrasonographic measures of diaphragm function (DTF, DT, DE, D-RSBI), as well as pulmonary patterns observed through lung ultrasound (LUS), with the outcomes of weaning.

## 2. Materials and Methods

### 2.1. Study Design and Setting

This observational, retrospective, single-center study was conducted in the Intensive Care Unit (ICU) of University Hospital Las Higueras, Talcahuano, Chile, from June 2020 to November 2020. The study adhered to the Strengthening the Reporting of Observational Studies in Epidemiology (STROBE) guidelines [12] (see Supplementary Materials Table S1) and complied with the ethical principles outlined in the World Medical Association (WMA) Declaration of Helsinki for medical research involving human subjects [13].

The study protocol received approval from the local ethics review board of the Servicio de Salud Metropolitano Oriente in the Santiago Metropolitan Region, Chile, under protocol number 5141/2021. Given the retrospective nature of the study and in accordance with local regulations regarding the use of previously collected data, the requirement for informed consent was waived.

### 2.2. Participants

All patients aged 18 years or older admitted to the Intensive Care Unit (ICU) with a diagnosis of COVID-19 who underwent invasive MV for at least 48 h and initiated the weaning process from mechanical ventilation were included in this study. Exclusion criteria included patients for whom ultrasound follow-up during the IMV weaning process was not feasible, those with a history of neuromuscular disease, and individuals with pre-existing chronic cardiorespiratory dysfunction.

### 2.3. Data Collection

Data were extracted from a retrospective registry and anonymized for analysis. Variables of interest included baseline characteristics such as age, sex, and medical history. ICU care and outcomes were recorded, including the duration of invasive MV, the duration of non-invasive MV (comprising conventional oxygen therapy [COT], high-flow oxygen therapy [HFOT], and non-invasive ventilation [NIV]), the presence of tracheostomy, extubation failure, ICU complications, length of ICU stay, and all-cause ICU mortality.

Ventilatory variables during the weaning process were assessed, including ventilatory mode, pressure support in cmH_2_O, positive end-expiratory pressure (PEEP) in cmH_2_O, fraction of inspired oxygen (FiO_2_) in percentage, arterial partial pressure of oxygen/FiO_2_ ratio (PaO_2_/FiO_2_), maximum inspiratory pressure (MIP), rapid shallow breathing index (RSBI), and peak expiratory flow (PEF).

Ultrasound parameters were also evaluated during the weaning process, including DTF, DT, DE, D-RSBI, and LUS findings before and after the spontaneous breathing trial (SBT).

### 2.4. Definitions

Maximum inspiratory pressure (MIP) measures the maximum pressure generated during inhalation against an obstructed airway and is used to assess inspiratory muscle strength as a predictor of weaning success [5,6,8,9,10,11,12,13,14].

The rapid shallow breathing index (RSBI) is the ratio of respiratory rate to tidal volume. It is calculated by dividing the respiratory frequency (measured in breaths/min) by tidal volume (measured in L) during a minute of spontaneous breathing [10].

Spontaneous breathing trial (SBT) failure is defined by any of the following criteria: a respiratory frequency greater than 35 breaths/min, poor respiratory mechanics, use of accessory muscles for more than 5 min, persistent peripheral oxygen saturation (SpO_2_) below 88%, a 25% change in heart rate and/or blood pressure from baseline [11].

Weaning failure (WF) is identified as the requirement for invasive MV following a successful SBT and extubation within the subsequent 48 h [15,16] or when the patient fails to complete the SBT. In contrast, successful weaning is characterized by the completion of an SBT with good tolerance and subsequent extubation without the need to reconnect to invasive mechanical ventilation within the following 48 h [16].

### 2.5. Local Protocol for Weaning from Mechanical Ventilation

Before initiation of invasive MV, all patients were treated for acute respiratory failure with conventional oxygen therapy, high-flow nasal cannula, or noninvasive ventilation. All patients ultimately required invasive MV due to the failure of noninvasive ventilation. Management adhered to the ARDS COVID-19 guidelines [17]. The weaning process started when attending physicians determined that patients were ready, considering they exhibited at least a minimum level of cooperation (S5Q 3/5). According to the “WIND classification”, the initiation of weaning was identified by a separation attempt, irrespective of prior reductions in ventilatory support. In intubated patients, a separation attempt from MV was defined as a SBT with or without extubation, or 24 h or more of spontaneous ventilation through a tracheostomy without assistance [10].

Patients eligible for weaning from MV were those who met the following criteria: assisted ventilation mode with FiO_2_ < 0.5, a PaO_2_/FiO_2_ ratio > 150 mmHg, a maximum PEEP of 10 cmH_2_O, and hemodynamic stability with norepinephrine requirements < 0.2 µg/kg/min. These patients were routinely assessed for the possibility of undergoing an SBT and subsequent extubation [18].

The protocolized SBT consisted of a period of 30 to 60 min under assisted MV with the following ventilatory setting: pressure support of 5 cmH_2_O and PEEP of 0 cmH_2_O [19]; before undergoing SBT, the following clinical ventilatory and ultrasound parameters were collected: maximal inspiratory pressure, peak expiratory flow (PFE), RSBI, DT, DFT, DE, D-RSBI, and LUS.

### 2.6. Ultrasound Evaluation

Ultrasonographic evaluations were performed using the Logiq-e R8 (GE HealthCare^®^, Chicago, IL, USA) ultrasound machine. Each assessment was conducted by experienced kinesiologists working in the intensive care unit, with an intra-class correlation coefficient (ICC) exceeding 0.95 for all measurements. Patients were positioned supine at a 30–45° angle for all ultrasonographic evaluations. Ultrasonographic evaluations were conducted while each patient was on pressure support ventilation. Lung ultrasound (LUS) was assessed both prior to and during the spontaneous breathing trial (SBT) to monitor changes in aeration throughout the test. The assessment before the SBT was performed with the patient on mechanical ventilation in pressure support mode, while the LUS during the SBT was conducted using the ventilatory settings mentioned above, in accordance with the local protocol.

Diaphragmatic thickness (DT) was measured using a linear transducer with frequencies ranging from 7.5 to 10 MHz in both B-mode and M-mode imaging. The transducer was placed in the zone of diaphragmatic apposition, located between the anterior axillary and midaxillary lines and between the eighth and tenth intercostal spaces. Measurements were taken during tidal breathing and/or maximal inspiratory effort. For this assessment, patients were on MV in pressure support mode, with ventilatory settings of 0 cmH_2_O PEEP, and a support pressure of 5 cmH_2_O, following the local SBT protocol. Diaphragmatic thickness during both expiration and inspiration was assessed to calculate the diaphragmatic thickening fraction (DTF) using the following formula [20]:

$$DTF=\frac{\left(EIT-EET\right)}{EET}\times 100$$

where EIT is end-inspiratory thickness, and EET is end-expiratory thickness of the diaphragm.

Diaphragmatic excursion (DE) was assessed using a high-frequency linear probe (>10 MHz) in M-mode. The measurement was taken as the amplitude of the wave, with the sector transducer placed under the right and left costal crests in the midclavicular line, aimed at visualizing the diaphragmatic dome. For this assessment, patients were on MV in pressure support mode and a ventilatory setting of 0 cmH_2_O PEEP and a support pressure of 5 cmH_2_O, following the local SBT protocol. The diaphragmatic rapid shallow breathing index (D-RSBI) was calculated using the formula: D-RSBI = Respiratory Frequency/DE [21].

LUS was performed using the eight-point method (Supplementary Materials Figure S1). The quadrants were defined vertically by the parasternal, right and left anterior axillary, and right and left posterior axillary lines, and horizontally by the clavicular, mammillary, and costal ridge lines. Each area was assigned a score from 0 to 3 based on pulmonary aeration [22], as follows:

- 0 points: Normal aeration with the presence of A-lines or fewer than 2 isolated B-lines.
- 1 point: B1 pattern with more than 3 comet tails and less than 50% coalescence in the ultrasound window, indicating moderate loss of pulmonary aeration with multiple well-defined B-lines.
- 2 points: B2 pattern with more than 50% coalescence in the ultrasound window, indicating severe loss of pulmonary aeration with multiple coalescent B-lines.
- 3 points: Pattern C, representing complete loss of pulmonary aeration.

The total LUS score ranged from 0 to 24 points.

### 2.7. Statistical Analysis

The sample size calculation was based on the findings of Spadaro et al. [21], who reported an incidence of weaning failure of 33%. To achieve a diagnostic accuracy with an area under the receiver operating characteristic (AUROC) curve of 0.8, a Type I error of 0.05, a power of 90%, and accounting for a 20% dropout rate, a minimum of 59 patients was required [11].

For statistical analysis, a descriptive analysis was first conducted, including estimates of the mean, median, standard deviation, and the 25th and 75th percentiles for quantitative variables, as well as the frequencies and relative frequencies for qualitative variables. The Shapiro–Wilk test was used to assess the normality of the quantitative data. When the Shapiro–Wilk test *p*-value was less than 0.05, indicating non-normal distribution, non-parametric methods were applied.

To assess differences between quantitative variables concerning weaning failure, both the parametric Student’s *t*-test and the non-parametric Mann–Whitney U-test were utilized. Student’s *t*-test was used to determine if there were significant differences between the means of two independent samples, applicable when data are normally distributed, and variances are approximately equal (homoscedasticity). The Mann–Whitney U-test was employed when data did not follow a normal distribution, comparing the medians of two independent samples.

For examining the association between qualitative variables, a Chi-square test was applied. This test evaluated whether there was a relationship between two nominal qualitative variables, determining if the relationship was dependent or independent. The Chi-square test compared observed frequencies with expected values to identify statistically significant differences.

The logistic regression model was initially applied in a bivariate context, involving the dependent variable (weaning failure) and one predictor variable at a time. This analysis was used to determine cutoff points (thresholds) based on the receiver operating characteristic (ROC) curve for categorizing these variables. Odds ratios (OR) and their 95% confidence intervals (CIs) for weaning failure were calculated using these categorized variables.

A multiple logistic regression model was then developed, incorporating the categorized variables with *p*-values less than 0.2 from the bivariate analysis as covariates. This model aimed to predict weaning failure by analyzing binary data and estimating the probability of an event occurring based on the independent variables.

A significance level of 5% was used for all analyses, which were conducted in the R 4.1.3 environment (R Core Team, Vienna, Austria, 2021).

## 3. Results

### 3.1. Baseline Characteristics of Patients

**Figure 1:**
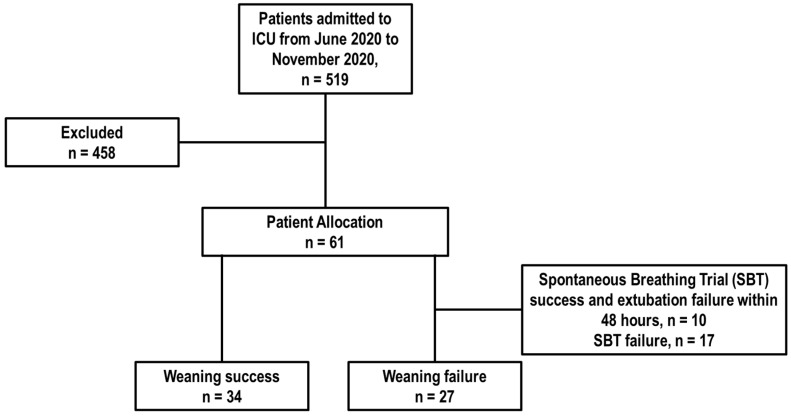
Patient flowchart: This chart illustrates the process of patient selection for the study.

During the study period, 519 patients were admitted to the ICU. Of these, 126 patients did not require MV, 92 were ventilated for less than 48 h, and 240 either did not have ultrasound follow-up or met exclusion criteria. Consequently, a total of 61 patients (Figure 1), who met the inclusion criteria, were analyzed.

The average age of these patients was 63 years (SD = 9.7), with 36% being female. The most prevalent comorbidity was hypertension (56%), followed by diabetes mellitus (44%).

The median SOFA (Sequential Organ Failure Assessment) score at ICU admission was 6 (IQR = 2–12). Patients who experienced weaning failure had a significantly higher median SOFA score compared to those who succeeded in weaning [weaning failure (WF) 9 (IQR = 2–12) vs. weaning success (WS) 3.5 (IQR = 2–11), *p* < 0.001].

Regarding the disease course, the median duration of illness before mechanical ventilation was 10.7 days (IQR = 3–35). Patients who experienced weaning failure had a significantly shorter median duration of illness before starting mechanical ventilation compared to those who succeeded in weaning [weaning failure (WF): 8.9 days (IQR = 3–28) vs. weaning success (WS): 12 days (IQR = 3–35), *p* = 0.040].

During their ICU stay, 9 patients (15%) experienced renal dysfunction, and 6 patients (10%) experienced cardiovascular dysfunction, with no significant differences between the weaning failure and weaning-success groups.

Throughout the period of mechanical ventilation, 32 patients (52%) required prone positioning. Notably, the number of patients requiring prone positioning during mechanical ventilation was significantly higher in the successful weaning group compared to the weaning-failure group [WF: 12 (44%) vs. WS: 20 (59%), *p* = 0.003].

Additionally, the mean duration of invasive MV and the duration of MV before the SBT were longer in the weaning-failure group compared to the success group (*p* < 0.001 for both).

The mean duration of COT was also longer in the weaning-failure group compared to the success group (*p* = 0.030), whereas the duration of HFOT was shorter in the weaning-failure group (*p* = 0.008).

The median length of ICU stay was 17 days (IQR = 3–65) and was significantly longer for the weaning-failure group compared to the success group [WF 30 (IQR = 7–65) vs. WS 10 (IQR = 3–51), *p* < 0.001]. The overall mortality rate was 9.8%, with a significantly higher rate observed in the weaning-failure group compared to the -success group [WF 22.2% vs. WS 0%, *p* = 0.014].

Detailed information on patients’ medical history, baseline characteristics, and outcomes, categorized by weaning failure or success, is provided in Table 1.

### 3.2. Characteristics of Weaning Parameters in Patients Who Failed of Succeeded in Weaning

All patients were mechanically ventilated via an endotracheal tube upon ICU admission. Weaning was unsuccessful in 27 patients (44.3%): 17 patients (27.9%) failed the SBT, and 10 patients (16.4%) required reintubation within 48 h. Of the 17 patients (27.9%) who subsequently underwent tracheostomy, 14 (82.4%) had previously failed the SBT, and 3 (30.0%) had previously been reintubated. Patients who failed weaning were ventilated for a significantly longer duration compared to those who succeeded (29 days vs. 7 days, *p* < 0.001).

Before mechanical ventilation (MV), patients received conventional oxygen therapy (COT), non-invasive ventilation (NIV), and high-flow oxygen therapy (HFOT). Among the patients who experienced weaning failure, COT was predominantly used in 17 patients (63%). Conversely, in the group that succeeded in weaning, HFOT was the predominant treatment used in 28 patients (82%).

For those who successfully completed a spontaneous breathing trial (SBT) and underwent extubation, the post-MV treatments included COT, NIV, or HFOT. In the successful weaning group, 5 patients (15%) utilized COT, 13 patients (38%) used NIV, and 16 patients (47%) were on HFOT (see Table 1).

The mean pressure support was 10.2 cmH_2_O (SD = 2.5) and was significantly higher in patients who failed weaning compared to those who succeeded [WF: 11.2 cmH_2_O (SD = 2.3) vs. WS: 9.4 cmH_2_O (SD = 2.5), *p* = 0.003]. Mean PEEP, FiO_2_, and blood gas analysis parameters did not differ significantly between the groups, except for SaO_2_, which was notably higher in the WS group compared to the WF group [WF: 94.5% (SD = 1.7) vs. WS: 95.3% (SD = 1.2), *p* = 0.035]. The ventilatory settings and blood gas analysis results before the SBT are summarized in Table 2.

The mean RSBI was 55.10 (SD = 24.93), and it was significantly higher in the WF group compared to the WS group [WF 69.53 (SD = 26.90) vs. WS 48.11 (SD = 20.97), *p* = 0.013]. The mean MIP was 42.10 (SD = 12.27) and was lower in the WF group than in the WS group [WF 36.32 (SD = 12.09) vs. WS 45.42 (SD = 11.24), *p* = 0.015]. The mean PFE, Right DTF, Left DTF, Right DE, Left DE, Right D-RSBI, and Left D-RSBI did not significantly differ between the weaning failure and success groups.

Right and left diaphragm thickness (DT) were significantly lower in the weaning-failure group compared to the -success group [Right DT: WF 0.20 (SD = 0.08) vs. WS 0.23 (SD = 0.06), *p* = 0.017; Left DT: WF 0.19 (SD = 0.11) vs. WS 0.22 (SD = 0.05), *p* = 0.005]. The inspiration thickness of the diaphragm was significantly lower in the weaning-failure group than in the -success group, but only in the Left diaphragm [WF 0.26 (SD = 0.22) vs. WS 0.30 (SD = 0.1), *p* = 0.006].

Lung ultrasound scores were significantly higher in the weaning-failure group compared to the success group, both before and after the SBT [Before SBT: WF 16.67 (SD = 4.74) vs. WS 13.76 (SD = 5.54), *p* = 0.019; After SBT: WF 19.50 (SD = 2.53) vs. WS 16.78 (SD = 5.41), *p* = 0.046] (Table 3).

### 3.3. Diagnostic Accuracy of Weaning Parameters for Predicting Weaning Failure

Bivariate logistic regression models were used to evaluate candidate variables for predicting weaning failure. The RSBI model revealed an odds ratio (OR) of 1.04 (95% CI 1.01–1.07, *p* = 0.010), indicating that the odds of weaning failure increase by 4% for each one-unit increase in the RSBI. The MIP model showed an OR of 0.92 (95% CI 0.86–0.99, *p* = 0.018), suggesting a reduced likelihood of weaning failure with each unit increase in MIP. The LUS score before SBT had an OR of 1.15 (95% CI 0.98–1.35, *p* = 0.025), reflecting a trend towards increased likelihood of weaning failure with higher LUS scores. Other parameters did not significantly predict weaning failure, as detailed in Table 4.

**Figure 2:**
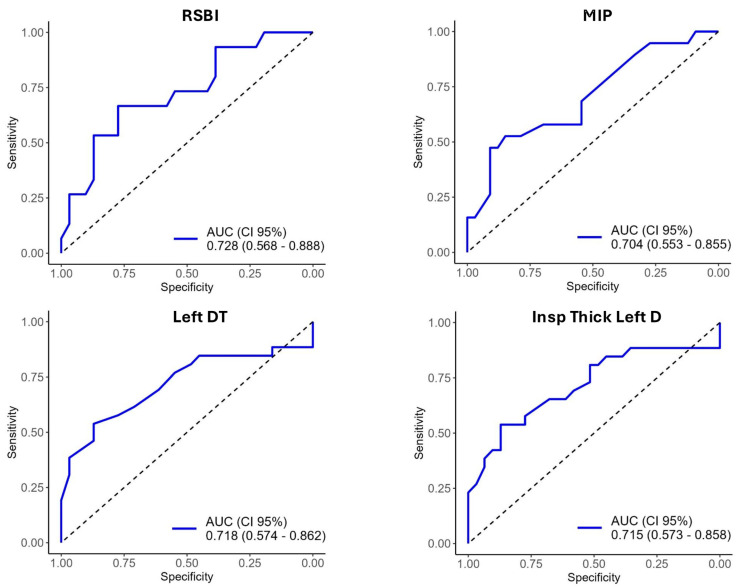
ROC Curves for Clinical And Ultrasonographic Variables. Figure Legend: RSBI, rapid shallow breathing index; MIP, maximal inspiratory pressure; PFE, peak expiratory flow; DTF, diaphragm inspiratory thickening fraction; DE, diaphragmatic excursion; D-RSBI, diaphragmatic rapid shallow breathing index; DT, diaphragmatic thickening; LUS, lung ultrasound.

The diagnostic accuracy of each variable was assessed using the area under the ROC curve (AUROC) and the corresponding thresholds. The RSBI exhibited an AUROC of 0.728, with an optimal threshold of 57.50 points. The diagnostic accuracy of all variables is presented in Supplementary Materials Table S2 and Figure 2.

### 3.4. Predictors of Weaning Failure

A comparison of weaning parameters, categorized based on their respective cutoff points, in relation to weaning outcomes, is presented in Supplementary Materials Table S3. In the multivariate logistic regression analysis, no variables were significantly associated with weaning failure, as detailed in Table 5.

## 4. Discussion

This observational single-center retrospective study described the characteristics and outcomes of weaning failure from MV in patients ventilated for ≥48 h due to acute respiratory failure related to COVID-19. We found that (1) weaning failed in 44.3% of patients. Among these, 27.9% failed the spontaneous breathing trial (SBT), 16.4% were re-intubated within 48 h, and 27.9% subsequently underwent tracheostomy; (2) patients who failed weaning had a significantly higher SOFA score at admission (9 vs. 3.5, *p* < 0.001), a shorter duration of symptoms before intubation (10.7 vs. 9.4 days, *p* = 0.040), longer MV duration (29 vs. 7 days, *p* < 0.001), longer ICU length of stay (34 vs. 14 days, *p* < 0.001), and higher mortality (22% vs. 0%, *p* = 0.014) compared to those who succeeded in weaning; (3) the weaning-failure group had significantly higher pressure support and lower SaO_2_ compared to the weaning-success group. However, PEEP, FiO_2_, and blood gas analysis parameters did not differ significantly between the groups; (4) among clinical and ultrasonographic indices, RSBI was significantly higher in the weaning-failure group, MIP was lower in the weaning-failure group, PFE, Right DTF, Left DTF, Right DE, Left DE, Right D-RSBI, and Left D-RSBI showed no significant differences between groups. Right and Left DT, and inspiration thickness of the left diaphragm were significantly lower in the weaning-failure group. Our study highlights the behavior of both hemi-diaphragms during the weaning process. Specifically, the evidence suggests that measurements of the right diaphragm can be extrapolated to reflect the behavior of the entire inspiratory muscle [10]. All measurements were conducted by specialized kinesiologists, achieving an intra-class correlation coefficient (ICC) greater than 0.95 across all analyses. This excellent correlation underscores the robustness of the ultrasound results presented in this observational study.

LUS was significantly higher in the weaning-failure group than in the -success group, both before and after SBT. Bivariate logistic regression identified RSBI, MIP, and LUS as being associated with weaning failure. However, multivariate logistic regression did not reveal any independent predictors of weaning failure. The disappearance of the association in the bivariate and multivariate analyses can be attributed to some factors: (1) Confounding Factors: Other clinical variables not measured in our study may influence the outcomes. For example, factors such as organ dysfunctions, or comorbidities could significantly impact weaning success or failure, potentially overshadowing the contribution of diaphragmatic function as assessed by ultrasound; (2) Variability in Measurements: diaphragmatic ultrasound is subject to high inter-operator variability. Although we provided extensive training for our kinesiologists, inherent variability might affect the reliability of the ultrasound measurements. This variability could contribute to the lack of significant association observed in subsequent analyses; and (3) Clinical Relevance of Non-sonographic Correlates: Our findings suggest that non-sonographic clinical indices, such as RSBI and MIP, may be equally or more valuable predictors of weaning failure than ultrasound parameters. This aligns with the existing literature that emphasizes the importance of traditional clinical measures in assessing respiratory muscle function and readiness for weaning [18,19]. In this context, the evidence indicates that further studies with larger patient populations are necessary to establish the precise role of ultrasonography in predicting the weaning process [2]. Hence, this study underscores the critical need to identify both clinical and ultrasonographic indices associated with weaning failure in critically ill COVID-19 patients. While previous research has primarily concentrated on biomarkers for predicting weaning failure, there has been limited exploration into the role of ultrasonography and conventional weaning parameters within this specific patient population. This study contributes valuable insights into how these indices can aid in predicting weaning outcomes and potentially improve management strategies in this challenging cohort [23,24,25]. The current study findings align with previous evidence, demonstrating that patients who experience weaning failure generally have higher comorbidities, more severe illness (as indicated by elevated SOFA scores), prolonged mechanical ventilation duration, and increased mortality rates. These factors are further exacerbated in the context of COVID-19, highlighting the severe impact of this disease on weaning outcomes [26,27]. The presence of multiple organ dysfunctions significantly impairs the body’s capacity to recover from acute illness, complicating the weaning process from mechanical ventilation. Prolonged weaning is often associated with complications such as neuromuscular weakness and diaphragmatic dysfunction, which further impede successful liberation from mechanical ventilation [28,29]. Additionally, COVID-19 induces systemic inflammatory responses marked by cytokine release and hyperinflammation, which can worsen organ damage, prolong ICU stays, and complicate both the weaning and recovery processes. The cumulative impact of these factors contributes to higher mortality rates in patients with elevated SOFA scores and significant comorbidities [26,27].

In our cohort, weaning failure occurred in 44.3% of patients, with approximately 28% failing the spontaneous breathing trial (SBT) and 16.4% requiring re-intubation within 48 h. The tracheostomy rate was about 28%, with 82.4% of tracheostomized patients having previously failed the SBT, and 30% of these individuals were re-intubated before undergoing tracheostomy. In comparison, the WEAN-SAFE study, the largest study on weaning from mechanical ventilation (MV), reported a weaning failure rate of approximately 35% [30]. The higher weaning failure rate in our cohort is likely attributable to the exclusive inclusion of COVID-19 patients, who typically require longer durations of mechanical ventilation compared to non-COVID-19 patients. This extended need for mechanical ventilation often complicates the weaning process and increases the likelihood of weaning failure [31,32]. Furthermore, the majority of COVID-19 patients in our study presented with severe acute respiratory distress syndrome (ARDS) and elevated SOFA scores, which reflect more complex and challenging weaning scenarios. These conditions are often associated with potential multi-organ dysfunction, further complicating the weaning process and contributing to the higher failure rates observed [26,27].

A global survey reported significant regional differences in the frequency of screening, methods used for SBTs, ventilator modes, care protocols, and the responsibilities of healthcare personnel involved in the weaning process from MV [33,34]. In our study, the weaning strategy employed was daily pressure support SBT with a gradual reduction in support over several days [19]. Evidence regarding the superiority of pressure support compared to T-piece ventilation in patients at high risk of extubation failure remains controversial [33,34]. In our cohort, the mean pressure support during SBT was approximately 10 cmH_2_O, and it was significantly higher in patients who failed weaning compared to those who succeeded (WF: 11 cmH_2_O vs. WS: 9 cmH_2_O, *p* = 0.003). This level of pressure support was sufficient to compensate for the inspiratory load [35], suggesting that the pressure support level was not the primary cause of weaning failure. Instead, other factors such as respiratory muscle weakness, impaired lung function, inadequate oxygenation, hypercapnia, sedation, psychological factors, infections, fluid overload, timing, and the overall condition of patients likely play a role [36]. This is supported by the observation that SaO_2_ was significantly lower in the weaning-failure group compared to the success group.

Our results indicate that the RSBI was significantly higher in patients who experienced weaning failure. The literature presents mixed evidence regarding RSBI’s predictive value for weaning success. Some recent studies suggest that while RSBI has moderate sensitivity, its specificity for predicting extubation success is poor [37]. Additionally, there has been a lack of consistent association between RSBI values and weaning success, regardless of the cutoff point used [38]. Conversely, our findings show that MIP was significantly higher in patients who successfully weaned from mechanical ventilation. This aligns with existing evidence that supports an association between higher MIP values and improved cough strength, which contributes to better mucociliary clearance following extubation [39].

In other cohorts of COVID-19 patients, the measurement of right hemi-diaphragmatic excursion has proven less useful in predicting weaning failure. However, lung ultrasonography scores have shown reliability in predicting weaning failure in critically ill COVID-19 patients [40]. Our study found that parameters such as PFE, RDTF, LDTF, RDE, LDE, RD-RSBI, and LD-RSBI did not differ significantly between weaning success and failure groups. This is consistent with another study which concluded that DTF was not predictive of weaning failure in COVID-19 patients [41]. Both right and left DT, but more interestingly, the left inspiration thickness of the diaphragm were significantly lower in patients who failed weaning compared to those who succeeded. This finding is novel, as the literature typically notes challenges in assessing the left hemidiaphragm due to the smaller acoustic window of the spleen and gas interposition in the stomach [42]. For this reason, previous studies have often undervalued this measure, and evidence addressing this question is scarce. However, given that the diaphragm is influenced by various anatomical and volumetric structures in the pulmonary region—particularly concerning the right hemidiaphragm—a targeted assessment becomes crucial for interpreting its functionality during ventilation. To address these challenges, we performed diaphragmatic ultrasound with patients in a supine position at a 30–45° angle and utilized experienced kinesiologists to achieve an intra-class correlation coefficient (ICC) > 0.95 for all tests. This approach highlights the value of bilateral diaphragmatic measurement, suggesting that while the right hemidiaphragm measurement is generally considered sufficient, including both sides can provide a more comprehensive assessment [10].

LUS was significantly higher in patients who experienced weaning failure, suggesting these patients were likely sicker. Consistent with this, evidence from a cohort of COVID-19 patients indicates that a LUS threshold of 10 points provides a specificity of 72.7% and sensitivity of 92.3% for predicting weaning failure [40]. LUS has proven to be a valuable tool for assessing pulmonary complications at the bedside, particularly during the COVID-19 pandemic. It played a critical role in identifying COVID-19 phenotypes with good correlation to computed tomography findings [43]. Additionally, LUS’s accessibility for bedside imaging minimized the need for patient transport to radiology facilities, thereby reducing the risk of airborne transmission associated with such transfers.

## 5. Limitations

This study has several limitations that should be acknowledged. First, the single-center design and small sample size limit the generalizability of our findings. Second, the absence of diaphragmatic and lung ultrasound measurements at ICU admission, along with the lack of repeated measurements, restricts our ability to provide a comprehensive assessment of muscular thickness and diaphragmatic and pulmonary involvement. Further research is needed to explore the relationship between ultrasound findings, mechanical ventilation variables, and muscular factors, accounting for daily variations. Third, as the study focused solely on patients with COVID-19, comparisons with other ICU populations are challenging, which may introduce selection bias. Fourth, the retrospective nature of the study limits our ability to control for confounding variables and their impact on weaning outcomes. Moreover, several important variables essential for understanding the study population—such as lung compliance during controlled mechanical ventilation, CT scan findings, fluid balance, and diuresis—could not be investigated, thereby limiting the interpretation of our findings. Fifth, the limited control over variables compared to a prospective study affects our ability to establish causality. Additionally, despite an intra-class correlation coefficient (ICC) > 0.95 for all measurements, some limitations of ultrasound must be noted, including subjective interpretations, the need for operator experience, and the presence of interposed air or gas that may limit visualization. Finally, the study design precludes establishing a temporal relationship between exposure and outcome.

## 6. Conclusions

Patients undergoing mechanical ventilation due to respiratory failure require a comprehensive approach to determine the optimal timing for weaning from ventilatory support. Evaluating respiratory mechanics, maximal muscle strength, and assessing lung parenchyma and diaphragm function are crucial strategies in this process. Our study underscores the importance of ultrasound, particularly in analyzing both the right and left hemidiaphragms. Although ultrasound indices alone did not show a definitive association with weaning failure, they provide valuable insights into diaphragmatic function and can enhance the overall assessment strategy. By integrating ultrasound into a multi-faceted evaluation, clinicians can better navigate the complexities of weaning from mechanical ventilation and potentially improve patient outcomes.

## Figures and Tables

**Table 1:** Baseline characteristics of patients and outcomes, in the overall cohort, and according to weaning failure and weaning success.

	Overall (*n* = 61)	Weaning Failure (*n* = 27)	Weaning Success (*n* = 34)	*p*-Value
Age, years, mean (SD)	63 (9.7)	64 (9.6)	62 (9.8)	0.351
Gender, *n* (%)				
Female	22 (36%)	12 (44.44%)	10 (29.41%)	0.344
Male	39 (64%)	15 (55.55%)	24 (70.59%)	
Comorbidities, *n* (%)				
Hypertension	34 (56%)	15 (56%)	19 (56%)	>0.99
Diabetes mellitus	27 (44%)	15 (56%)	12 (35%)	0.186
Renal failure	5 (8%)	2 (7%)	3 (9%)	>0.99
Pulmonary disease	0 (0%)	0 (0%)	0 (0%)	-
Neurological disease	0 (0%)	0 (0%)	0 (0%)	-
Malignancy	0 (0%)	0 (0%)	0 (0%)	-
SOFA score, points, median (IQR)	6 (2–12)	9 (2–12)	3.5 (2–11)	<0.001
History of symptoms				
Days of illness before hospital admission, median (IQR)	6.4 (1–26)	5.5 (2–11)	7.0 (1–26)	0.215
Days of illness before ICU admission, median (IQR)	8.9 (1–33)	7.6 (3–22)	10 (1–33)	0.086
Days of illness before IMV, median (IQR)	10.7 (3–35)	8.9 (3–28)	12 (3–35)	0.040
Treatment before IMV, *n* (%)				
COT	22 (36%)	17 (63%)	5 (15%)	0.000
NIV	2 (3%)	1 (4%)	1 (3%)	
HFOT	37 (61%)	9 (33%)	28 (82%)	
Treatment after IMV, *n* (%)	*n* = 44	*n* = 10	*n* = 34	
COT	5 (11%)	0 (0%)	5 (15%)	0.418
NIV	21 (48%)	5 (50%)	13 (38%)	
HFOT	18 (41%)	5 (50%)	16 (47%)	
Prone vigilant before the IMV, *n* (%)	24 (39%)	10 (37%)	14 (41%)	0.742
Prone during the IMV, *n* (%)	32 (52%)	12 (44%)	20 (59%)	0.003
Organic dysfunction during ICU stay, *n* (%)				
Renal	9 (15%)	5 (19%)	4 (12%)	0.460
Cardiovascular	6 (10%)	4 (15%)	2 (6%)	0.245
Hepatic	0 (0%)	0 (0%)	0 (0%)	-
Hematology	0 (0%)	0 (0%)	0 (0%)	-
Neurological	0 (0%)	0 (0%)	0 (0%)	-
Outcomes				
IMV duration, days, median (IQR)	10 (2–65)	29 (5–65)	5 (2–15)	<0.001
IMV duration before SBT, days, median (IQR)	7 (2–36)	14 (2–36)	5 (2–15)	<0.001
COT duration, days, median (IQR)	0 (0–6)	0 (0–4)	1.5 (0–6)	0.030
NIV duration, days, median (IQR)	0 (0–7)	0 (0–7)	0 (0–7)	0.864
HFOT duration, days, median (IQR)	1 (0–6)	0 (0–4)	0 (0–6)	0.008
ICU—length of stay, days, median (IQR)	17 (3–65)	10 (3–51)	30 (7–65)	<0.001
ICU—mortality, *n* (%)	6.0 (9.8%)	6.0 (22.2%)	0 (0%)	0.014

IMV = invasive mechanical ventilation, SOFA = Sequential Organ Failure Assessment, ICU = intensive care unit; COT = conventional oxygen therapy; NIV = non-invasive ventilation; HFOT = high flow oxygen therapy. The Chi-square test or the Mann–Whitney U test was used to assess differences between groups, as appropriate. IQR = interquartile range (Min–Max), SD = standard deviation.

**Table 2:** Ventilatory settings, D-dimer levels and gas exchange characteristics before spontaneous breathing trial.

	Overall (*n* = 61)	Weaning Failure (*n* = 27)	Weaning Success (*n* = 34)	*p*-Value
Ventilatory setting before SBT				
Pressure support, cmH_2_O, mean (SD)	10.2 (2.5)	11.2 (2.3)	9.4 (2.5)	0.003
PEEP, cmH_2_O, mean (SD)	6.7 (1.5)	6.8 (1.4)	6.6 (1.6)	0.764
FiO_2_, %, mean (SD)	0.3 (0.5)	0.3 (0.5)	0.3 (0.5)	0.094
D-dimer, mean (SD)	1197 (1293)	1240 (1343)	1162 (1270)	0.835
Blood gas analysis before SBT				
pHa, mean (SD)	7.41 (0.04)	7.40 (0.05)	7.41 (0.04)	0.560
PaO_2_, mmHg, mean (SD)	77.5 (9.3)	76.5 (10.8)	78.3 (8.1)	0.309
PaO_2_/FiO_2_, mmHg, mean (SD)	248.4 (45.4)	235.6 (45.1)	258.5 (43.7)	0.058
PaCO_2_, mmHg, mean (SD)	41.4 (6.3)	42.0 (8.1)	40.9 (4.5)	0.850
HCO_3_^−^, mmol/L, mean (SD)	25.6 (3.1)	25.7 (4.0)	25.4 (2.1)	0.850
SaO_2_, %, mean (SD)	94.9 (1.5)	94.5 (1.7)	95.3 (1.2)	0.035

PEEP, positive end-expiratory pressure; FiO_2_, fraction of inspired oxygen; PaO_2_, arterial partial pressure of oxygen; PaCO_2_, arterial partial pressure of carbon dioxide; HCO_3_^−^, bicarbonate; SaO_2_, arterial saturation of oxygen. Student *t*-test or Mann–Whitney U-test was used as appropriate.

**Table 3:** Clinical and ultrasonographic indices of weaning failure and success in the overall cohort and by weaning outcome.

	Overall (*n* = 61)	Weaning Failure (*n* = 27)	Weaning Success (*n* = 34)	*p*-Value
RSBI	55.10 (24.93)	69.53 (26.90)	48.11 (20.97)	0.013
MIP	42.10 (12.27)	36.32 (12.09)	45.42 (11.24)	0.015
PFE	99.13 (27.32)	103.64 (31.07)	97.21 (25.85)	0.500
Right DTF	0.21 (0.25)	0.23 (0.31)	0.18 (0.19)	0.500
Left DTF	0.33 (0.36)	0.30 (0.46)	0.36 (0.26)	0.061
Right DE	1.75 (0.77)	1.73 (0.77)	1.77 (0.78)	0.900
Left DE	1.76 (0.72)	1.80 (0.85)	1.72 (0.60)	0.700
Right D-RSBI	1.83 (1.58)	2.27 (2.12)	1.49 (0.83)	0.300
Left D-RSBI	1.54 (0.88)	1.62 (0.96)	1.48 (0.83)	0.800
Right DT	0.22 (0.07)	0.20 (0.08)	0.23 (0.06)	0.017
Left DT	0.21 (0.08)	0.19 (0.11)	0.22 (0.05)	0.005
Inspiration thickness of the right diaphragm	0.26 (0.08)	0.24 (0.09)	0.27 (0.08)	0.160
Inspiration thickness of the left diaphragm	0.28 (0.17)	0.26 (0.22)	0.30 (0.1)	0.006
LUS before SBT	15.05 (4.81)	16.67 (4.74)	13.76 (4.54)	0.019
LUS after SBT	17.61 (4.86)	19.50 (2.53)	16.78 (5.41)	0.046

RSBI, rapid shallow breathing index; MIP, maximal inspiratory pressure; PFE, peak expiratory flow; DTF, diaphragm inspiratory thickening fraction; DE, diaphragmatic excursion; D-RSBI, diaphragmatic rapid shallow breathing index; DT, diaphragmatic thickening; LUS, lung ultrasound. Student’s *t*-test or the Mann–Whitney U test was used as appropriate.

**Table 4:** Bivariate logistic regression models for predicting weaning failure: evaluation of candidate variables.

Variable	Odds Ratio	95% CI	*p*-Value
RSBI	1.04	1.01–1.07	0.010
MIP	0.92	0.86–0.99	0.018
PFE	1.01	0.99–1.03	0.458
Right DTF	2.37	0.29–19.18	0.418
Left DTF	0.58	0.12–2.73	0.494
Right DE	0.92	0.46–1.85	0.825
Left DE	1.18	0.48–2.89	0.720
Right D-RSBI	1.43	0.94–2.19	0.098
Left D-RSBI	1.20	0.57–2.55	0.631
Right DT	0.001	<0.001–5.11	0.116
Left DT	0.003	<0.001–6.14	0.136
Inspiration thickness of the right diaphragm	0.01	<0.001–8.63	0.190
Inspiration thickness of the left diaphragm	0.15	0.004–6.29	0.318
LUS before SBT	1.16	1.02–1.31	0.025
LUS after SBT	1.15	0.98–1.35	0.096

RSBI, rapid shallow breathing index; MIP, maximal inspiratory pressure; PFE, peak expiratory flow; DTF, diaphragm inspiratory thickening fraction; DE, diaphragmatic excursion; D-RSBI, diaphragmatic rapid shallow breathing index; DT, diaphragmatic thickening; LUS, lung ultrasound.

**Table 5:** Multivariate logistic regression models for predicting weaning failure.

Variables	Mod. 1 *p*-Value	Mod. 2 *p*-Value	Mod. 3 *p*-Value	Mod. 4 *p*-Value	Mod. 5 *p*-Value	Mod. 6 *p*-Value	Mod. 7 *p*-Value	Mod. 8 *p*-Value	Mod. 9 *p*-Value	Mod. 10 *p*-Value	Mod. 11 *p*-Value
(Intercept)	1.000	0.997	0.997	0.996	0.997	0.996	0.994	0.995	0.002	0.002	0.001
RSBI (>57.50)	1.000	0.701	0.701	0.597	0.876	0.945	0.509	0.569	0.190	0.174	
MIP (>36.00)	1.000	0.999	0.999	0.998							
PFE (>117.50)	1.000	0.997	0.997	0.996	0.997	0.996	0.996	0.070	0.089	0.061	0.051
Right DTF (>0.305)	1.000	0.999	0.999								
Left DTF (>0.194)	1.000										
Right D-RSBI (>1.765)	1.000	0.917	0.917	0.792	0.876	0.806	0.934	0.944	0.458		
Right DT (>0.195)	1.000	0.997	0.997	0.996	0.997	0.996	0.996				
Left DT (>0.195)	1.000	0.997	0.997	0.997	0.997	0.997					
inspiration thickness of the left diaphragm (>0.205)	1.000	1.000									
LUS before SBT (>15.50)	1.000	0.998	0.998	0.998	0.998						
LUS after SBT (>15.50)	1.000	0.997	0.997	0.997	0.997	0.996	0.995	0.995			
AIC	24.00	32.99	30.99	29.49	28.48	27.52	30.06	30.39	32.26	30.80	30.66

RSBI, rapid shallow breathing index; MIP, maximal inspiratory pressure; PFE, peak expiratory flow; DTF, diaphragm inspiratory thickening fraction; D-RSBI, diaphragmatic rapid shallow breathing index; DT, diaphragmatic thickening; LUS, lung ultrasound. The models use the traditional input method, initially with all variables and removing the least significant one (or the one with the smallest coefficient), with weaning failure as the dependent variable.

## Data Availability

Data are available at the corresponding author under reasonable request.

## Supplementary Materials

The following supporting information can be downloaded at: https://www.mdpi.com/article/10.3390/diagnostics14202263/s1, Table S1. Strengthening the Reporting of Observational Studies in Epidemiology (STROBE) Statement—Checklist of Items that Should be Included in Reports of Cohort Studies; Figure S1. Lung Ultrasound Score According to the Eight-Points Method; Table S2. Area Under the Curve (AUC) and Threshold (Cutoff Point) of Predictor Variables in Relation to the Outcome; Table S3. Comparison Between Variables Referring to Weaning Parameters. Categorized Using the Cutoff Point. in Relation to Weaning Outcome.
