# Supplementary material for: Predictive Value of Diaphragm and Lung Ultrasonography for Weaning Failure in Critically Ill Patients with Acute Respiratory Failure Due to COVID-19 Pneumonia
Source: Diagnostics (Basel). 2024 Oct 11;14(20):2263. doi: 10.3390/diagnostics14202263 (PMC11505932; doi:10.3390/diagnostics14202263)
Supplement: Supplementary file 1 [file diagnostics-14-02263-s001.zip › diagnostics-3228827-supplementary.pdf]

Supplementary Materials (SM)

Table of Contents

- **Table S1.** Strengthening the Reporting of Observational Studies in Epidemiology (STROBE) Statement—Checklist of Items that Should be Included in Reports of Cohort Studies. [Page 2,3]
- **Figure S1.** Lung Ultrasound Score According to the Eight-Points Method. [Page 4]
- **Table S2.** Area Under the Curve (AUC) and Threshold (Cutoff Point) of Predictor Variables in Relation to the Outcome. [Page 5]
- **Table S3.** Comparison Between Variables Referring to Weaning Parameters. Categorized Using the Cutoff Point. in Relation to Weaning Outcome. [Page 6]

Table S1. The STROBE (Strengthening the Reporting of Observational Studies in Epidemiology) Statement provides a checklist for reporting observational studies. including cohort studies

	Item No	Recommendation	Page
**Title and abstract**	1	(*a*) Indicate the study's design with a commonly used term in the title or the abstract	1
		(*b*) Provide in the abstract an informative and balanced summary of what was done and what was found	2
**Introduction**			
Background/rationale	2	Explain the scientific background and rationale for the investigation being reported	3
Objectives	3	State specific objectives. including any prespecified hypotheses	4
**Methods**			
Study design	4	Present key elements of study design early in the paper	4
Setting	5	Describe the setting. locations. and relevant dates. including periods of recruitment. exposure. follow-up. and data collection	4
Participants	6	(*a*) Give the eligibility criteria. and the sources and methods of selection of participants. Describe methods of follow-up	4
		(*b*) For matched studies. give matching criteria and number of exposed and unexposed	-
Variables	7	Clearly define all outcomes. exposures. predictors. potential confounders. and effect modifiers. Give diagnostic criteria. if applicable	5.6
Data sources/ measurement	8*	For each variable of interest. give sources of data and details of methods of assessment (measurement). Describe comparability of assessment methods if there is more than one group	5.6
Bias	9	Describe any efforts to address potential sources of bias	5.6
Study size	10	Explain how the study size was arrived at	7
Quantitative variables	11	Explain how quantitative variables were handled in the analyses. If applicable. describe which groupings were chosen and why	
Statistical methods	12	(*a*) Describe all statistical methods. including those used to control for confounding	7
		(*b*) Describe any methods used to examine subgroups and interactions	7
		(*c*) Explain how missing data were addressed	7
		(*d*) If applicable. explain how loss to follow-up was addressed	
		(*e*) Describe any sensitivity analyses	7
**Results**			

Participants	13*	(a) Report numbers of individuals at each stage of study—eg numbers potentially eligible. examined for eligibility. confirmed eligible. included in the study. completing follow-up. and analysed	8
		(b) Give reasons for non-participation at each stage	8
		(c) Consider use of a flow diagram	8
Descriptive data	14*	(a) Give characteristics of study participants (eg demographic. clinical. social) and information on exposures and potential confounders	9
		(b) Indicate number of participants with missing data for each variable of interest	9
		(c) Summarise follow-up time (eg. average and total amount)	9
Outcome data	15*	Report numbers of outcome events or summary measures over time	10.11
Main results	16	(*a*) Give unadjusted estimates and. if applicable. confounder-adjusted estimates and their precision (eg. 95% confidence interval). Make clear which confounders were adjusted for and why they were included	12.13
		(*b*) Report category boundaries when continuous variables were categorized	
		(*c*) If relevant. consider translating estimates of relative risk into absolute risk for a meaningful time period	
Other analyses	17	Report other analyses done—eg analyses of subgroups and interactions. and sensitivity analyses	12.13
**Discussion**			
Key results	18	Summarise key results with reference to study objectives	13
Limitations	19	Discuss limitations of the study. taking into account sources of potential bias or imprecision. Discuss both direction and magnitude of any potential bias	15
Interpretation	20	Give a cautious overall interpretation of results considering objectives. limitations. multiplicity of analyses. results from similar studies. and other relevant evidence	14
Generalisability	21	Discuss the generalisability (external validity) of the study results	14
**Other information**			
Funding	22	Give the source of funding and the role of the funders for the present study and. if applicable. for the original study on which the present article is based	

Figure S1.

Lung ultrasound score according to the eight-points method

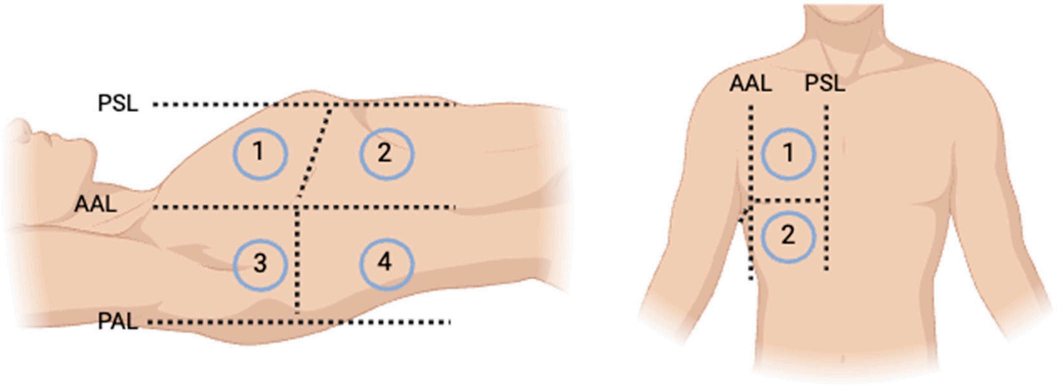

The thoracic wall was segmented into eight distinct areas for scanning. with one scan performed for each. These areas included two anterior and two lateral regions on each side. The anterior thoracic wall was divided from the parasternal line (PSL) to the anterior axillary line (AAL) into upper and lower halves—extending from the clavicle to the nipple line. and from the nipple line to the diaphragm. The lateral thoracic wall was segmented from the anterior axillary line to the posterior axillary line (PAL) into upper and basal halves.

Table S2. AUC (Area Under Curve) and Threshold (Cutoff point) of the predictor variables in relation to the outcome.

Variables	AUC	Threshold
RSBI	0.728	57.50
MIP	0.704	36.00
PFE	0.562	117.50
Right DTF	0.548	0.305
Left DTF	0.646	0.194
Right DE	0.514	1.925
Left DE	0.525	1.80
Right D-RSBI	0.580	1.765
Left D-RSBI	0.524	1.190
Right DT	0.679	0.195
Left DT	0.718	0.165
Inspiration thickness of the right diaphragm	0.606	1.75
Inspiration thickness of the left diaphragm	0.715	2.05
LUS before SBT	0.685	15.50
LUS after SBT	0.686	15.50

Legend: RSBI. rapid shallow breathing index; MIP. maximal inspiratory pressure; PFE. peak expiratory flow; DTF. diaphragm inspiratory thickening fraction; DE. diaphragmatic excursion; D-RSBI. diaphragmatic rapid shallow breathing index; DT. diaphragmatic thickening; LUS. lung ultrasound.

Table S3. Comparison between the variables referring to weaning parameters. categorized using the cutoff point. in relation to the Weaning outcome.

Variables		Weaning Success N	Success %	Weaning Failure N	Failure %	OR	CI (95%)	p-value*
RSBI	<= 57.50	24	82.76	5	17.24	REF	-	-
	> 57.50	7	41.18	10	58.82	6.86	1.75 – 26.83	0.004
MIP	<= 36.00	3	25.00	9	75.00	REF	-	-
	> 36.00	30	75.00	10	25.00	0.11	0.03 – 0.49	0.002
PFE	<= 117.50	27	77.14	8	22.86	REF	-	-
	> 117.50	6	50.00	6	50.00	3.38	0.85 – 13.41	0.076
Right DTF	<= 0.305	27	64.29	15	35.71	REF	-	-
	> 0.305	7	36.84	12	63.16	3.09	1.00 – 9.51	0.046
Left DTF	<= 0.194	6	28.57	15	71.43	REF	-	-
	> 0.194	25	69.44	11	30.56	0.18	0.05 – 0.57	0.003
Right DE	<= 1.925	23	60.53	15	39.47	REF	-	-
	> 1.925	8	44.44	10	55.56	1.92	0.62 – 5.96	0.258
Left DE	<= 1.80	14	63.64	8	36.36	REF	-	-
	> 1.80	9	47.37	10	52.63	1.94	0.56 – 6.80	0.295
Right D-RSBI	<= 1.765	24	63.16	14	36.84	REF	-	-
	> 1.765	7	41.18	10	58.82	2.45	0.76 – 7.89	0.129
Left D-RSBI	<= 1.190	11	61.11	7	38.89	REF	-	-
	> 1.190	9	47.37	10	52.63	1.75	0.47 – 6.45	0.402
Right DT	<= 0.195	9	33.33	18	66.67	REF	-	-
	> 0.195	25	73.53	9	26.47	0.18	0.06 – 0.54	0.002
Left DT	<= 0.195	4	22.22	14	77.78	REF	-	-
	> 0.195	27	69.23	12	30.77	0.13	0.03 – 0.47	0.001
inspiration thickness of the right diaphragm	<= 0.205	8	44.44	10	55.56	REF	-	-
	> 0.205	26	60.47	17	39.53	0.52	0.17 – 1.59	0.251
inspiration thickness of the left diaphragm	<= 0.205	4	22.22	14	77.78	REF	-	-
	> 0.205	27	69.23	12	30.77	0.13	0.03 – 0.47	0.001
LUS before SBT	<= 15.50	21	72.41	8	27.59	REF	-	-
	> 15.50	13	40.62	19	59.38	3.84	1.31 – 11.27	0.013
LUS after SBT	<= 15.50	11	100.00	0	0.00	REF	-	-
	> 15.50	21	60.00	14	40.00	-	-	0.012

Legend: RSBI. rapid shallow breathing index; MIP. maximal inspiratory pressure; PFE. peak expiratory flow; DTF. diaphragm inspiratory thickening fraction; DE. diaphragmatic excursion; D-RSBI. diaphragmatic rapid shallow breathing index; DT. diaphragmatic thickening; LUS. lung ultrasound. *Chi-square test; N = Number of observations; %= relative frequency; OR = Odds Ratio; CI = Confidence interval.
